# Transfusion with Tested Bloods

**Published:** 1918-04

**Authors:** 


### BLOOD TRANSFUSION ABSTRACTS

***Transfusion with Tested Bloods***. Howard T. Karsner, M. D. (Cleveland), Captain, M. R. C. Abs. from the *Journal of the American Medical Association*, March 16, 1918.

Accidents due to the use of indiscriminately selected donors are more frequent in base hospitals than in those nearer the front, for the reason, perhaps, that patients at the base have been wounded for a longer period and have been drained by infection to a greater degree.

Two methods of testing blood have been used in the author's laboratory : Kolmer's method, which is satisfactory and accurate, but which causes a delay of 2 1/2 hours that might be serious in a case of severe hemorrhage; and Lee's method, based on the fact that the interaction of serum and red corpuscles places every individual in one of four groups [2]. The Lee method has been used with great success.

A large number of donors may be grouped in the fashion indicated by Lee provided they are free from any infectious disease.

1. For details of the experiments and clinical experience, see B. M. J., March 2, 1918.
2. See p. 458.

Although, according to Lee, Group 4 donors may be used for any transfusion and Group 1 may receive blood from any donor, the author, because of one unfortunate experience, prefers to have donor and recipient in the same group when possible. He believes that the protection of the recipient's cells from agglutination by the donor's plasma is probably the result of an antiagglutinative body rather than of dilution, for it can be shown that serum retains its agglutinative properties when diluted as much as thirty-two times. Naturally such an anti-agglutinative body would operate better against a diluted plasma than against an undiluted plasma.

The problem is further complicated by the variability of cells from different individuals. The patient worn down by infection may possibly be less resistant to such agglutinative sera than the freshly wounded man.

*Testing at Advanced Hospitals.* — It is obviously impossible to test recipient's blood when immediate transfusion is necessary and no laboratory is near. It is possible, however, to have prospective donors tested and to use Group 4 as the universal donor.

For purposes of testing at the casualty clearing stations the "resuscitation teams" are now being provided with sera in tubes, similar to those in which glycerinized vaccine virus is supplied. The serum may be preserved with 0.5 per cent. carbolic acid, which does not alter the titer of the serum, and which agglutinates powerfully for at least a month after being phenolated, having remained at room temperature in the meantime. The following equipment is easily transportable :

Six microscopic slides.

Six tubes, 75 by 10 mm.

Fifty small tubes of Group 2 serum.

Fifty small tubes of Group 3 serum.

One copper wire loop (platinum and aluminium not being available).

One hand lens of 10 × magnification.

The serum is blown from the tubes directly on to the slide, and an equal amount of blood is suspended in physiologic sodium chlorid solution, rubbed in with the loop. The slide is inverted, stands at room temperature for ten minutes, and the result is read with the hand lens. The loop must be dried with a towel or blotter in going from one serum to the other, but does not necessarily need sterilization. This method, when employed by experienced persons, has been found to be entirely satisfactory.

